# Transcription Factors of the bHLH Family Delineate Vertebrate Landmarks in the Nervous System of a Simple Chordate

**DOI:** 10.3390/genes11111262

**Published:** 2020-10-26

**Authors:** Lenny J. Negrón-Piñeiro, Yushi Wu, Anna Di Gregorio

**Affiliations:** Department of Molecular Pathobiology, New York University College of Dentistry, 345 E 24th Street, New York, NY 10010, USA; lnp260@nyu.edu (L.J.N.-P.); yw1406@nyu.edu (Y.W.)

**Keywords:** ascidian, bHLH, *Ciona*, CNS, epiphysis, hypophysis, hypothalamus, nervous system, notochord, sensory vesicle

## Abstract

Tunicates are marine invertebrates whose tadpole-like larvae feature a highly simplified version of the chordate body plan. Similar to their distant vertebrate relatives, tunicate larvae develop a regionalized central nervous system and form distinct neural structures, which include a rostral sensory vesicle, a motor ganglion, and a caudal nerve cord. The sensory vesicle contains a photoreceptive complex and a statocyst, and based on the comparable expression patterns of evolutionarily conserved marker genes, it is believed to include proto-hypothalamic and proto-retinal territories. The evolutionarily conserved molecular fingerprints of these landmarks of the vertebrate brain consist of genes encoding for different transcription factors, and of the gene batteries that they control, and include several members of the bHLH family. Here we review the complement of bHLH genes present in the streamlined genome of the tunicate *Ciona robusta* and their current classification, and summarize recent studies on proneural bHLH transcription factors and their expression territories. We discuss the possible roles of bHLH genes in establishing the molecular compartmentalization of the enticing nervous system of this unassuming chordate.

## 1. Introduction

Vertebrata, Tunicata (or Urochordata), and Cephalochordata are the three clades of the phylum Chordata. Along with the other members of the subphylum Tunicata, ascidians occupy a unique evolutionary position, as they are considered the extant taxon closest to vertebrates [1]. Since the beginning of the past century, studies on ascidians have provided a valuable reference for chordate development, first by informing comparative biological studies between embryos featuring different ontogenetic strategies [2,3,4,5], and, more recently, by shedding light on the molecular mechanisms underlying a variety of developmental processes, both ancestral and derived [6,7,8,9,10]. These processes include formation of the notochord [11,12,13] neural tube closure and dorsoventral patterning [14,15], regionalization of the central nervous system (CNS) [16,17], heart development [18,19], formation of the cardiopharyngeal precursors [20,21,22], biosynthesis and role of thyroid hormones [23], and specialization of the digestive tract [24,25]. The search for compartments evolutionarily related to the anatomical structures that punctuate the vertebrate brain in invertebrate chordates stretches over several decades, and started out with comparative morphological observations and immunocytochemical studies, which have been complemented, in more recent years, by phylogenomic analyses and single-cell molecular fingerprinting. These recent investigations, several of which have been mainly focused on species belonging to the cosmopolitan genus *Ciona*, have revealed that the developmental programs of these structures rely upon a relatively streamlined molecular machinery that shares numerous homologies with its vertebrate counterparts [26,27,28,29,30,31]. Thus, studies in ascidians have been tracing back the origins of anatomical and physiological structures that emerged in these simple chordates in an uncomplicated form and have been elaborated upon by increasingly complex vertebrates.

Basic helix-loop-helix (bHLH) genes represent an ancient superfamily of transcription factors (TFs) that are widely represented in eukaryotes; they are found in plants, where they are involved in processes ranging from flower pigmentation to iron homeostasis [32,33], in fungi, where they control hyphal growth, melanin production, and virulence [21], and in all metazoan phyla analyzed thus far [34,35]. The evolutionary history of the bHLH superfamily has been reconstructed through molecular phylogenetic studies and comparative genomic analyses, and it is believed to have involved a diversification event during the Pre-Cambrian era, at the time when early metazoans appeared, and a subsequent expansion of the family before the split of bilaterians and cnidarians [36]. Members of the bHLH superfamily often form homodimers, and they can also heterodimerize with other bHLH proteins. These interactions influence their respective effects on transcriptional regulation, and enable them to function as either activators or repressors of gene expression in different spatial and temporal contexts [34]. It has been hypothesized that increasingly complex interactions between bHLH TFs have mediated their functional transition from ancestral regulators of cell division to coordinators of tissue differentiation [37]. Throughout metazoan phyla, members of the bHLH family of TFs regulate a variety of processes, including cell-lineage specification, cell differentiation, response to environmental stress, and maintenance of circadian rhythm, among others; in particular, bHLH TFs are critical components of the gene regulatory cascades underlying myogenesis and neurogenesis [37,38,39,40]. Consequently, their mutations and deregulation are responsible for developmental defects and diseases, and contribute to tumorigenesis and metastasis [40,41,42].

Here we focus on the roles of bHLH TFs in the specification and morphogenesis of nervous structures during chordate development. One reason for their widespread functions in vertebrate neurogenesis is that, during embryogenesis, bHLH TFs are expressed in crucial organizing centers of the nervous system, the *zona limitans intrathalamica* (ZLI) and the midbrain-hindbrain boundary (MHB, also known as isthmic organizer), where they participate in pre-patterning morphogenetic events [43,44]. Later on, bHLH genes participate in the differentiation of anatomo-physiological landmarks of the vertebrate CNS, such as hypophysis, hypothalamus and retinal territories, epiphysis, and habenulae, where they are involved in the differentiation of specific neuronal subtypes, hormone-producing cells, establishment of the circadian rhythm, and generation of behavioral outputs [45]. As most evolutionarily conserved bHLH genes are present in single copies in the *Ciona* genome, studies on their roles in the development and compartmentalization of the straightforward ascidian CNS can inform related investigations in more complex chordates, and simplify the interpretation of the phenotypes resulting from their inactivation.

## 2. bHLH Transcription Factors and Organizing Centers in the Developing Ascidian Nervous System

**Figure 1:**
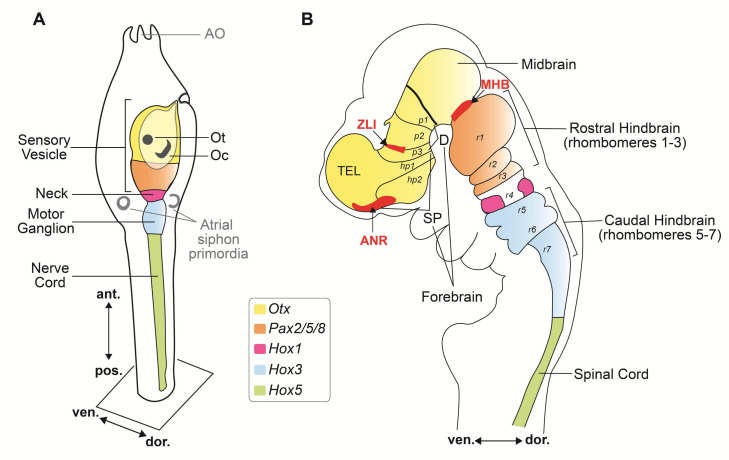
General organization and presumed homologous regions of the central nervous system (CNS) in ascidian larvae and vertebrate embryos. Drawings of a *Ciona* larva (**A**), ~18 h after fertilization and a mouse embryo (**B**), ~9 days old (stage E9.5). The developing nervous systems of these divergent chordates exhibit a comparable anterior-posterior sequential expression of the genes *Otx* (yellow), *Pax2/5/8* (orange), *Hox1* (magenta), *Hox3* (blue), and *Hox5* (green). Abbreviations: ANR, anterior neural ridge; ant., anterior; AO, adhesive organ; D, diencephalon; dor., dorsal; hp, hypothalamic prosomere; MHB, mid-hindbrain boundary; Oc, ocellus; Ot, otolith; p, prosomere; pos., posterior; r, rhombomere; SP, secondary prosencephalon; TEL, telencephalon; ven., ventral; ZLI, *zona limitans intrathalamica*. Adapted from [10,17,55].

After fertilization, ascidian embryos develop into tadpole-like larvae within less than one day, and after swimming around for several hours, they rapidly metamorphose into juveniles and slowly continue to grow into adults. The simple chordate body plan of the ascidian larva contains only ~2600 cells, organized into a few main embryonic tissues, among which a notochord, located in the center of the tail, and a modest yet fascinating CNS located rostral and dorsal to it [46,47,48]. The notochord, the eponymous feature of the phylum, is invariantly composed of only 40 cells, and expresses homologs of *Brachyury*, *Foxa2*, and other evolutionarily conserved transcriptional regulators, resembling the more sophisticated vertebrate notochords [12,28,49,50,51,52]. In addition, genes of the homeobox family, including *Otx*, *Hox1*, *Hox3*, and *Hox5*, are expressed in the ascidian larval CNS in a rostrocaudal pattern comparable to that seen in developing vertebrate embryos [16,53,54]. These comparative studies have provided evidence that the simple CNS of the ascidian larva (Figure 1A), which consists of only 177 neurons and ~123 accessory cells, exhibits a topological organization relatable to that of the three primary brain vesicles of vertebrates: forebrain (prosencephalon), midbrain (mesencephalon), and hindbrain (rhombencephalon) (Figure 1B). In vertebrates, the developing forebrain is subdivided into diencephalon and secondary prosencephalon. The diencephalic territories delineated as prosomeres 1, 2, and 3 (p1–p3; Figure 1B), give rise to the pretectum, thalamus, and prethalamus, respectively (Figure 1B). The secondary prosencephalon is further subdivided into the caudal and rostral hypothalamic prosomeres (hp1 and hp2, respectively; Figure 1B), which encompass the presumptive hypothalamic region, telencephalon, and optic vesicles [55,56,57]. The formation of these structures, in vertebrates, is orchestrated by organizers, which act as signaling centers and as local sources of fibroblast growth factor (FGF) and other instructive molecules able to induce cell divisions and early patterning events [58,59]; these organizing centers also express TFs of both the homeobox and bHLH families, among others. Interestingly, the evolutionary origins of vertebrate organizing centers have been traced back to the acorn worms, members of the phylum Hemichordata, whose non-chordate embryos contain regions that display gene expression patterns homologous to those seen in the anterior neural ridge (ANR), ZLI and MHB [60]. In ascidian embryos, a region equivalent to the ZLI is yet to be identified, even though the genomes of *Halocynthia roretzi* and other ascidians seem to contain bHLH genes related to *Olig3*, a ZLI marker [44], whose expression, however, remains to be elucidated. The existence of an ascidian MHB with organizing properties related to those observed in vertebrate embryos had been initially ruled out on the basis of the expression pattern of the MHB marker *Dmbx* [61]. However, subsequent studies have uncovered the dynamic expression patterns of *Ciona Dmbx* and *Hox* genes in the larval CNS, and have provided initial clues on its subtly compartmentalized structure [62]. The identification in the motor ganglion of developing *Ciona* tadpoles of a localized source of the *Ciona* ortholog of Fgf8 (*Ciona* Fgf8/17/18), an evolutionarily conserved mediator of the MHB organizer activity, and the reported role of *Ciona* Fgf8/17/18 in the specification of the neck region of the larval CNS, are currently considered indicative that a simplified form of the MHB with organizer-like features is indeed present in the larval CNS of *Ciona*, and is responsible for its rostro-caudal regionalization [63]. A gene related to a bHLH TF that characterizes the MHB in *Xenopus*, *Hes-related1* (*XHR1*) [43], is present in the *Ciona* genome (*Ciona Hey*, Table 1), however its expression was not detected at any of the stages analyzed by whole-mount in situ hybridization (WMISH) before metamorphosis [50].

Another organizing center required for the development of neural structures is the prechordal plate, a mesendodermal embryonic territory anterior to the notochord. In particular, the prechordal plate is required for the formation of the hypothalamus [64], and in zebrafish, it expresses a member of the *Id3* bHLH subfamily [65]. A physically distinguishable structure homologous to the prechordal plate seems to be absent in ascidians.

## 3. Anatomo-Physiological Vertebrate Landmarks in the Uncomplicated Nervous System of the Ascidian Larva

**Figure 2:**
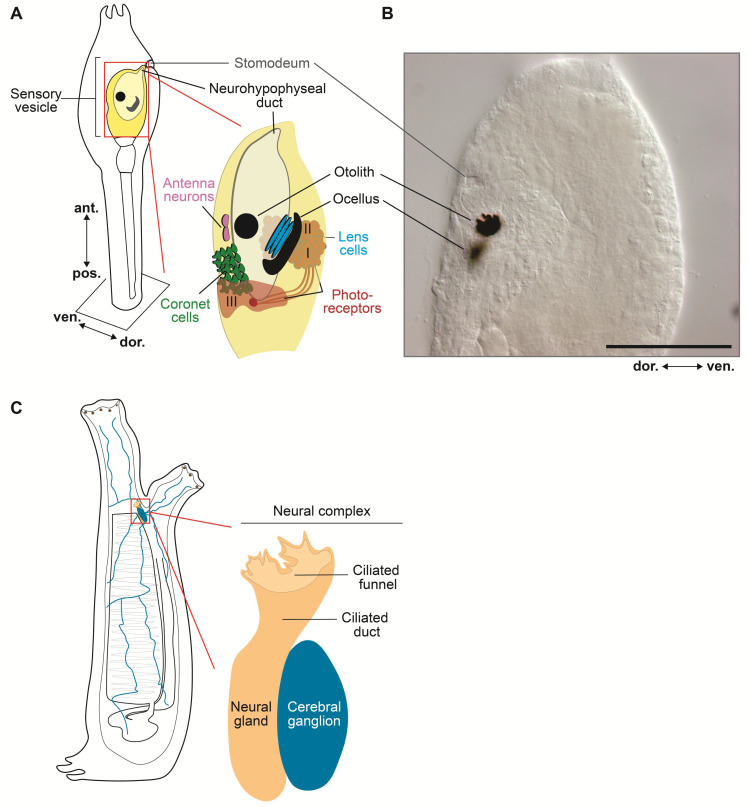
Larval sensory vesicle and adult neural complex of *Ciona*. (**A**) Schematic view of the sensory vesicle, the ‘brain’ of the ascidian larva, its sensory organs, and the primordia of the hypophysis and stomodeum. On the right side of the sensory vesicle reside the pigmented ocellus and the associated lens cells and photoreceptors (group I and II), whereas the left side contains the otolith, antenna neurons, coronet cells, and photoreceptor cells (group III). (**B**) Microphotograph of the trunk of a *Ciona* larva, showing the developing stomodeum, the otolith, and the ocellus. Scale bar: 25 µm. (**C**) Schematic view of the sessile filter-feeder adult, highlighting the neural complex, located between the two siphons, and its components, the cerebral ganglion and the neural gland. Nerve fibers from the neural complex (blue) innervate multiple organs and tissues. Adapted from [48,85,86].

In vertebrates, the forebrain develops dorsally into the telencephalon and ventrally into the diencephalon [56,57]. The telencephalic-derived cerebral cortex appeared in lower vertebrates and increased vastly in size and functional complexity in amniotes. Its development relies upon a delicate balance between the action of proneural bHLH TFs, such as Mouse Achaete-scute homolog 1 (Mash1), Neurogenin 1, and Neurogenin 2, which promote differentiation of cortical precursor cells, and the function of proliferation-promoting bHLH TFs of the Id and Hes subfamilies, which maintain these cells in an undifferentiated, multipotent state [66]. Differently from the cortex, the diencephalic-derived structures, hypothalamus, hypophysis, and epithalamus, are believed to have originated before the divergence of the three chordate subphyla. In the case of the hypothalamus, this hypothesis is based on the comparable regionalization of the homeobox genes *Otp*, *Nkx2.1* and *Meis* between the vertebrate hypothalamic primordium, the anterior-ventral region of the sensory vesicle (SV; also known as brain vesicle) of *Ciona* (Figure 2A), and the anterior-most region of the CNS of the amphioxus *B. lanceolatum* [29,30,55]. The anteroventral territory of the ascidian larval CNS, which develops into a portion of the SV, contains dopamine-synthesizing cells that resemble the clusters of dopaminergic cells in the developing hypothalamus of vertebrates [29], and the presence of dopamine-synthesizing cells in the anterior-most region of the CNS is also observed in amphioxus [67]. Together, these findings support the idea that tunicates and cephalochordates inherited a proto-hypothalamus from a common chordate ancestor. Dorsal to the SV, in the ascidian larva, resides an ectoderm-derived domain that forms the stomodeum (Figure 2A,B), which is considered equivalent to a vertebrate placode [68] and gives rise to the incurrent oral siphon of the adult ascidian (Figure 2C). The stomodeum expresses the homeobox genes *Pitx* and *Six3/6*, similarly to all chordate mouth primordia examined to date [69,70].

In amphioxus, a derivative of Hatschek’s left diverticulum, the ciliated pit of the preoral organ, is considered the homolog of the vertebrate adenohypophysis (anterior pituitary gland) [71]. This area expresses the evolutionarily conserved pituitary-specific marker Pit-1/POU1F1, which encodes a POU-homeodomain TF [72,73], as well as *Pitx* (*pituitary homeobox*), a paired-type homeodomain TF that acts downstream of the Cerberus/Nodal/Lefty gene cascade in the establishment of left-right asymmetry [74]. A bona fide homolog of *Pit-1/POU1F1*, which is required for proper pituitary development in higher vertebrates, seems to be missing from the *Ciona* genome [75]. Nevertheless, territories related to the vertebrate hypophysis have been identified in both the larval and the adult ascidian body plan. At the beginning of the swimming larva stage, the ectoderm-derived neurohypophyseal duct, which protrudes from the SV, makes contact with the wall of the stomodeum, and expresses *Pitx*, similarly to the vertebrate adenohypophysis primordium [68,75,76,77,78,79]. Shortly after, the lumen of the neurohypophyseal duct coalesces with that of the stomodeum, while still in contact with the lumen of the SV, at a time when the stomodeum is still plugged by the transparent tunic that covers the larval body (Figure 2A) [68]. By the late larval stages, the connection of the neurohypophyseal duct with the lumen of the SV is lost, and both the duct and the stomodeum open into the endodermally-derived pharynx [68]. At metamorphosis, the neurohypophyseal duct gives rise to the neural gland, which remains connected to the pharynx through a ciliated duct and a ciliated funnel (Figure 2C). The neural gland had originally been suggested as the adenohypophysis homolog, on the basis of the immunohystochemical properties of some of its cells [80]; however, subsequent studies in *Ciona* have related to the vertebrate adenohypophysis the ciliated funnel, because it expresses *Pitx* and derives from the oral ectoderm of the stomodeum [68,75,81]. Notably, TFs of the Pitx family have been shown to physically interact with ubiquitously expressed group A bHLH proteins (Table 2), and to synergize with them in the transcriptional regulation of their downstream genes during pituitary development in mice [82]. In particular, mouse Pitx1 is recruited to promoter regions by DNA-bound bHLH TFs NeuroD1 and Pan1 [82]. Together with three other proneural bHLH TFs, Neurogenin, Mash1, and Mouse Atonal homolog 3 (Math3), NeuroD1 controls hypophyseal differentiation in mice, and participates in the functional specification of hormone-secreting cells that compose the adenohypophysis [83]. In *Ciona*, a gene related to *NeuroD*, *NeuroD-like* (Table 1), is expressed in epidermal sensory neurons, in the anterior SV and in the motor ganglion [84]; however, its possible overlap with *Ciona* genes related to *Achaete-scute* and *Atonal* has not been ascertained.

Another anatomical point of reference of the vertebrate CNS, the epithalamus, which consists of the epiphysis (aka pineal gland, or pineal eye) and the two habenulae, relies on bHLH TFs for its formation [87,88]. Structures related to the vertebrate epithalamus have been identified in the simple nervous systems of cephalochordates and tunicates. In amphioxus, the lamellar body, a ciliary photoreceptor, is considered a presumptive epiphysis [89,90]. Even though ascidians lack an organized epiphysis, the *Ciona* larval CNS expresses *Noto*, a homeodomain TF that in zebrafish controls expression of two other bHLH TFs required for epiphyseal neurogenesis, Neurogenin 1 (ngn1) and Achaete-scute homolog 1a (ash1a) [91]. *Ciona Noto* is expressed in the posterior notochord and in the SV of tailbud embryos, where it is detected anteriorly to the otolith and the ocellus, and in between these sensory organs, where it is expressed at lower levels [92]. The single-copy *Ciona Neurogenin* (*Ci-Neurog*) is expressed in several small areas of the SV (see below), and one of the *Achaete-scute-like* genes (Table 1), is expressed in a small region of the anterior-ventral SV [50]. In addition, it has been proposed that the ocellus might represent a structure homologous to the epiphysis [93], which is also known as median eye, rather than to the lateral eyes of vertebrates, based on its shadow response, which in larvae of *Xenopus* is controlled by the epiphysis [93,94]. In vertebrates, the epiphysis and the suprachiasmatic nucleus control the circadian rhythm of the entire organism and its ability to adjust its metabolism to environmental changes, and bHLH-PAS type TFs expressed in these structures are in large part responsible for these functions [95]. Even though the *C. robusta* genome lacks clear orthologs of the bHLH circadian regulators, *Clock*, *Period*, *Dec1* and *Bmal1*, several genes show a nearly circadian rhythmic expression [96].

## 4. Cellular and Molecular Topography of the *Ciona* Larval Nervous System

After hatching from the protective chorion in which they develop for approximately 18 h, the lecithotrophic ascidian larvae begin to swim around, in search of a submerged substrate where they will settle, metamorphose, and start their adult life as sessile filter-feeders [10]. About 1.5 h after hatching, the larvae begin to exhibit intermittent tail flicks and spontaneous swimming, and develop a shadow response, being stimulated to swim by sudden reductions in light intensity [97]. In addition, they exhibit negative gravitaxis, and swim towards the surface of the water, while later on they start swimming away from the surface of the water in search of a dimly lit substrate, presumably hidden from possible predators, to which they will attach and spend the rest of their life [98] this latter behavior is originated by a negative phototactic response [99]. In addition to gravitaxis and phototaxis, ascidian larvae are capable of chemotaxis and mechanosensory responses (thigmotaxis) and can enter a state of sensory arousal [98]. Remarkably, swimming *Ciona* larvae can be sensitized through recurring variations in luminous stimuli, and can even show habituation and short-term retention of their responses [100,101]. The larval CNS is responsible for coordinating sensory processing, and for orchestrating the transition from the stereotypic free-swimming behavior of the hatched larvae to their commitment to find the substrate that will serve as their definitive home [102]. The ascidian larval CNS comprises four main structures; the hollow SV and the motor ganglion, also known as visceral ganglion, which are connected by a narrow neck, reside within the trunk (Figure 1A), while the tail contains the nerve cord (NC), which is composed by non-nervous accessory cells, known as ‘ependymal’ cells, and by the axons of neurons located in the motor ganglion [48]. The cholinergic motor neurons of this ganglion require for their specification a bHLH TF, the single-copy *Ciona* Ebf, which is also sufficient to elicit cholinergic characteristics when ectopically expressed in non-cholinergic neurons (Table 1) [103].

Together, these compartments contain approximately 330 cells, among which are 177 neurons that fall into several different subtypes based on their morphology and connectivity [27,48]. The simple compartments of the larval CNS of *Ciona* display anterior-to-posterior regionalized expression of the homeobox genes *Otx*, *Pax2/5/8*, and *Hox1* comparable to those observed in the vertebrate forebrain, midbrain, and hindbrain (Figure 1) [16,27,31,48,97,104]. In addition to the CNS neurons, the ascidian larva contains two pairs of bipolar tail neurons, which are considered homologous to the neurons of the dorsal root ganglia of vertebrates and whose bodies are located between the nerve cord and the tail epidermis [105], and several peripheral sensory neurons, scattered throughout the epidermis and organized into small groups in the papillae of the adhesive organ (Figure 1A) [106,107].

Similar to the brain of vertebrates, cell types and structures in the larval SV of ascidians display a distinct left/right side asymmetry, namely a right-sided ocellus and left-sided coronet cells (Figure 2A), which suggests that asymmetric CNS features may have appeared early during chordate evolution [48]. The larval SV is considered the most complex structure of the ascidian CNS, and is composed of two conspicuous melanin-pigmented sensory systems, the otolith (also called statocyst) and the ocellus (Figure 2A), which respond to Earth’s gravitational field and light, respectively [17,48,102,108,109,110]. The otolith is a unicellular organ. This nearly spherical cell contains a large intracellular pigmented granule that protrudes into the SV cavity and is anchored to the ventral wall of the SV by a L-shaped foot [111,112]. The otolith is associated to a pair of ciliated cells and afferent glutamatergic antenna sensory neurons connected to relay neurons that project, across the neck, to the motor ganglion; together, these structures constitute the fairly simple gravitactic circuit of the ascidian larva [102]. The ocellus is a multicellular organ constituted by three components: one cup-shaped pigment cell, three lens cells, and about 30 photoreceptor cells, which are divided into three different groups based on their morphology and their location within the SV [113]. The photoreceptors of groups I and II are associated to the pigment cell located at the right dorsal side of the SV, while those of group III are located ventro-medially and constitute the non-pigmented ocellus, whose function is yet to be ascertained [112,114,115]. The photoreceptor cells also express a vertebrate-type opsin, Ci-opsin1, and Ci-arrestin; another opsin, Ci-opsin3, is expressed throughout the entire SV [93,101,114]. Both function and formation of the ocellus depend upon the evolutionarily conserved homeodomain TF Retinal homeobox (Rx), and its inactivation via morpholino oligonucleotide (MO) microinjection impairs both formation and function of this structure [116,117]. On the other hand, a bHLH TF, Mitf, has been shown to be essential for the formation of melanin-synthesizing pigment cells in vertebrates [118], and in the ascidian *H. roretzi* the ectopic expression of this gene is sufficient to induce the expression of genes necessary for melanogenesis [119]. Of note, the pigment cells of the ascidian SV are considered homologous to the vertebrate melanocytes, which are neural crest derivatives and thus represent an additional attribute shared by ascidians and vertebrates [120]. Interestingly, repression of the melanogenetic activity of *Mitf* in the ocellus precursors by FoxD is responsible for the reduced pigmentation of this photoreceptor structure [120]; a similar molecular mechanism is employed in zebrafish embryos to suppress Mitf-dependent melanogenesis in the neural-crest derived light-reflecting iridophores [121].

Another bHLH TF involved in neural crest cells migration, localization, and differentiation in vertebrate embryos is Twist, which is required, in particular, for the specification of both cephalic and cardiac neural crest cells [122,123]. Interestingly, the *Ciona* genome contains three copies of *‘Twist-like’* genes, likely resulting from a lineage-specific duplication, and they are all expressed in the mesenchyme, the pluripotent group of cells that remain relatively undifferentiated during embryonic development and after metamorphosis give rise to several adult structures, including body wall muscle, blood cells, and cells of the cellulose-based tunic [10,50]. Reportedly, the bHLH domains of Twist-r.a and Twist-r.b are identical, while their C-terminal regions are divergent [124]. Of these three *Twist-like* genes, *Twist-r.b* (Table 1) is the closest to human *TWIST1*, and when ectopically expressed in the progenitors of the pigment cells (a9.49 lineage) [125] is able to reprogram these cells, which are normally stationary, into migrating neural-crest-like cells [120].

Within the presumed hypothalamic region of the SV, described above, resides a group of cells that express tyrosine hydroxylase (TH), the enzyme involved in the rate-limiting step of the biosynthesis of dopamine and other catecholamines. These TH-expressing cells include different cell types, among which a subpopulation of coronet cells and at least one neuronal subtype [29]. The coronet cells, which send bulbous protrusions into the cavity of the SV, are located anteriorly to the group III photoreceptors, on the left side of the ventral wall of the SV (Figure 2A) [48]. The role of coronet cells in ascidians is still debated; nonetheless, based on the expression of TFs and other molecular fingerprints, such as components of the catecholamines synthesis pathway, they are considered related to the amacrine dopaminergic cells of the vertebrate retina [29,109]. Through functional studies, the *Ciona* coronet cells were revealed capable of accumulating serotonin [126]. Together with the reported expression in the SV of other markers of the hypothalamus and retina of vertebrates, such as *Six3/6*, *Rx*, *Meis*, *Pax6*, and visual cycle proteins, these findings support the idea that the ascidian CNS may have inherited a proto-retinal territory from a chordate ancestor [126,127]. The posterior-most region of the SV contains in its left dorsal side one large interneuron, the eminens cell, which projects directly to the proximal tail, and in its ventral side two interneurons, whose projections arch dorsally before extending towards the tail (large ventroposterior sensory interneurons [106,112]. All these neurons, as well as the recently identified additional eminens neurons, are considered GABAergic [97,112,128]. Single-cell transcriptional profiling has determined that eminens neurons express several bHLH genes, including *Ebf, Bhlh-tun2, Max,* and *Neurog* [27] (Table 1).

## 5. bHLH Transcription Factors in the Nervous System of Adult Ascidians

At metamorphosis, ascidian larvae lose some of the defining features of the chordate body plan, however they develop another chordate staple, the pharyngeal slits, in addition to a functional digestive tract, a tubular heart, and a primitive thyroid, the endostyle [129,130]. Along with the notochord and the muscles flanking it, the larval nerve cord disappears during tail retraction. However, while most of the larval neurons are lost, the non-neural ependymal cells act as pluripotent stem cells and give rise to most of the neurons of the adult CNS [131]. A small number of neurons derive from the delamination of the neurohypophyseal duct [68], and localize to the anterior tip of the ciliated funnel (Figure 2C) [131]. The adult nervous system consists of the neural gland, which derives from the neurohypophyseal duct, and the cerebral ganglion, which is mainly formed through the transdifferentiation of the larval ependymal cells [131]. Despite their transition from motile to sessile life forms, and the massive remodeling of their nervous system, which includes the loss of their photoreceptors, adult ascidians are still capable of responding to light stimuli. In particular, variable light stimuli can induce adult ascidians to contract their siphons and to spawn their gametes [132]. These responses have been attributed to the pigmented spots around the openings of the siphons, which are tentatively regarded as photoreceptors (Figure 2C) [133], and to the presence of retinal proteins and light-sensitive neurons in the cerebral ganglion [134,135] (Figure 2C). WMISH studies have revealed the nearly ubiquitous expression of *Hif*, *Myc*, and *ARNT* in the neural gland, and in numerous other structures of *Ciona* juveniles (Table 1) [136]. Instead, *Hes.b* exhibits a more localized expression in young adults, being expressed in the body wall muscle and in the stigmatal cells of the branchial sac, the site of a stem cell niche whose descendants contribute to the regeneration of the oral siphon [137]. Expression of *AHR* in *Ciona* juveniles was not detectable by WMISH, which suggests that this gene may not be expressed at this stage [136]. The expression patterns and the roles of the other bHLH genes in young adults remain to be characterized.

## 6. The bHLH Family of Transcription Factors in *Ciona*

*Ciona robusta* (previously *C. intestinalis* type A) [138] features one of the most compact chordate genomes (~120 Mb), estimated to contain 14,072 genes [139,140], of which nearly 400 encode for TFs [50]. Several large-scale studies using WMISH analysis and MO-mediated gene knockdowns have elucidated the expression patterns and the functions of numerous genes, and have outlined their roles in the development of the embryonic tissues of *Ciona* [50,136,141,142,143,144,145]. In addition, a large-scale study has identified the consensus binding sites for several *Ciona* TFs [146].

**Figure 3:**
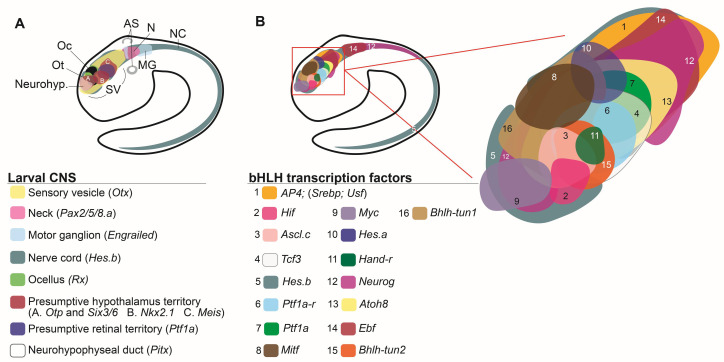
The panoply of bHLH genes expressed in the modest CNS of the *Ciona* larva. (**A**) The four main subdivisions of the larval CNS, as delineated by the expression patterns of *Otx* (sensory vesicle) *Pax2/5/8.a* (neck), *Engrailed* (motor ganglion), and *Hes.b* (expressed in all previous subdivisions, and in the nerve cord). The region of the sensory vesicle posited to represent a proto-hypothalamus-retinal territory is delineated by the expression of *Otp, Six3/6, Nkx2.1*, *Meis,* and *Ptf1a*. The territory that gives rise to the photoreceptors associated to the ocellus is marked by the expression of *Rx*, and the neurohypophyseal primordium is labeled by the expression of *Pitx*. Adapted from [62]. (**B**) The palette of bHLH genes expressed in the *Ciona* SV and the complex tapestry that they delineate within this simple chordate brain. Each electronic brushstroke symbolizes the approximate expression pattern of a different bHLH gene, reconstructed from published WMISH expression analysis at mid/late-tailbud stage, according to the color/number code below this panel. The expression territories of *Tcf3*, *Id.b* and *Mnt-r* have not been included because the results of their respective WMISH were unclear. Adapted from: [29,50,84,108,120,145,163,164,165,166]. Abbreviations: AS, atrial siphon primordium; MG, motor ganglion; N, neck; NC, nerve cord; Neurohyp., neurohypophyseal primordium; Oc, ocellus; Ot, otolith; SV, sensory vesicle.

The number of genes encoding for bHLH proteins varies across species; the *Drosophila* genome contains 59 bHLH genes, while in humans the number of these genes has expanded to approximately 125 [36,147]. Of the 46 bHLH genes identified in the genome of *Ciona robusta*, 41 have been analyzed using WMISH and/or single-cell transcriptomic analyses; among these, at least 21 are expressed within the SV, the ascidian ‘brain’, during the tailbud stages (Table 1) [50], and are the main subject of this discussion (Figure 3). The bHLH domain consists of ~60 amino acids, organized into a cluster of conserved basic amino acid residues adjacent to two amphipathic α-helices, separated by a non-helical loop [148,149,150]. The bHLH domain mediates both the binding to DNA and the formation of either homodimers or heterodimers between different family members [148,149,151]. bHLH TFs belonging to different groups recognize different hexanucleotide sequences, referred to as E-box sequences (generic consensus sequence: CANNTG), and depending on the dimerization complexes that they form, they can act as either activators or repressors of transcription [148,152]. Initially, bHLH TFs were classified into different groups based on a combination of parameters, including their tissue distribution, affinity to DNA, and dimerization potential [150]. Later on, another classification method, based on phylogenetic relationships, presence of additional structural domains, and E-box binding affinity, has categorized bHLH TFs into six major groups (Table 2A–F) [148,150,151]. Hence, the 46 *C. robusta* genes that encode for *bona fide* bHLH TFs have been assigned to these six different groups (Table 2) [147], as discussed in detail hereinafter.

It is noteworthy that despite the compact size and relative simplicity of the *Ciona* genome, several lineage-specific duplications have been detected in the case of different families of transcriptional regulators, including the homeobox [153,154], the T-box [155], and the bHLH family as well. The genome of *Ciona robusta* contains three copies of *Twist* genes, but only one myogenic bHLH gene, *Ci-Mrf*, related to the four vertebrate myogenic factors, MyoD1, Myf5, MRF4, and myogenin [156], in addition to eliciting muscle differentiation, *Ciona* Mrf is able to suppress both notochord and endoderm development when ectopically expressed in these tissues [157]. In contrast, the ancestral *Mrf* gene has independently expanded in the amphioxus genome [158]. Both *C. savignyi* and *C. robusta* possess a single copy of *Mesp*, which has been shown to be necessary for the specification of the tubular heart that forms shortly after metamorphosis [159,160,161], as opposed to *Mesp1* and *Mesp2* genes found in vertebrates, which are responsible for heart progenitors specification and somitogenesis, respectively, and are often functionally redundant [162]. 

### 6.1. Group A

Group A bHLH genes are characterized by their ability to recognize and bind the E-box consensus sequences CAGCTG or CACCTG, either as homo- or heterodimers [148,150]. At least six *Ciona* group A bHLH TFs are expressed at the tailbud stage within the SV territory, namely *Ptf1a, Ptf1a-r, Tcf3, Atoh8, Ascl.c*, and *Neurogenin* (Figure 3B).

In vertebrates, *Ptf1a*, *HEB*, and *E2A*, the counterparts of Ptf1a, Ptf1a-r, and Tcf3, associate to form the heterotrimeric PTF1 complex. Ptf1a and HEB constitute the two DNA-binding subunits of the PTF1 complex, while E2A is required to import the complex into the nucleus [167]. Potential components of the PTF1 complex have been identified in the genome of *C. robusta. Ci-Ptf1a* (Ciona pancreas-associated transcription factor 1a) is exclusively expressed in the TH-expressing coronet cells of the SV at the tailbud and larval stages [126,165]. The territory of expression of *Ci-Ptf1a-r* (Ciona pancreas associated transcription factor 1a-related) at the mid/late tailbud stages is nearly overlapping with that of *Ci-Ptf1a* (Figure 3B). The precise territory of expression of *Ci-Tcf3* (*Ciona transcription factor 3*) is not very clear, however it seems that this gene might be expressed at low levels throughout the SV, and more intensely in the territory that overlaps with the expression domain shared by *Ci-Ptf1a* and *Ci-Ptf1a-r* [50], which would suggest that a complex homologous to the vertebrate PTF1 complex might exist in *Ciona* (Figure 3B).

MO-mediated loss of function of Ci-Ptf1a obliterated coronet cells development, while its overexpression resulted in the formation of supernumerary coronet cells, indicating that *Ci-Ptf1a* is indispensable for the specification of this cell type [165]. In vertebrates, Ptf1a is required for the specification and differentiation of both amacrine and horizontal cells of the retina, and, in particular, of the sub-population that synthesizes the neurotransmitter γ aminobutyric acid (GABA) [168,169]; in addition, Ptf1a is required for the development of GABAergic neurons in the dorsal horns of the spinal cord and for the differentiation of pancreatic progenitors [170,171].

*Ciona* Neurog is another bHLH TF of group A expressed within the SV at the tailbud stages [50]. The expression of *Neurog* initiates at the gastrula stage in precursors of the lateral ependymal cells of the nerve cord, and persists throughout embryonic development in the anterior nerve cord and in several areas of the SV [50]. Within the SV, Neurog is expressed in the territory that gives rise to several interneuron subtypes, including bipolar interneurons, coronet-associated ciliated SV interneurons, ciliated brain vesicle interneurons, and others [50,86]. In the tail, Neurog is necessary and sufficient for the specification, delamination, migration, and differentiation of the bipolar tail neurons (BTNs) [105]. BTNs are proposed as homologs of vertebrate dorsal root ganglia (DRG) neurons, based on the expression of *Neurog* in these cell types, their morphogenesis, their developmental origin from neural crest-like cells, and their role in relaying peripheral sensory information to the CNS [108]. The overexpression of *Neurog* resulted in the formation of supernumerary BTNs that recapitulated the stereotyped behaviors of neural crest cells [105]. A transcriptome profiling of BTNs determined that, in these cells, Neurog influences the expression of 698 genes out of 11,777 analyzed. Of the 76 Neurog-downstream targets that were further analyzed by in situ hybridization, 49 were confirmed to be expressed in BTNs, and 24 of them were found to be expressed within the SV as well [108]. Among them is *Bhlh-tun2* (Table 1). MO-mediated knockdown of Neurog resulted in the down-regulation of the zinc-finger protein *Myt1,* of *Fgf8/17/18*, and of *Delta-like* in the trunk lateral cells, which are the precursors of blood cells, longitudinal muscle, and oral siphon muscle of the adult [108]. In addition, the knockdown of *Neurog* resulted in the slight up-regulation of *Neurog* transcripts, which might indicate a negative autoregulatory feedback [164]. In vertebrates, the single-copy *Neurog* found in *Ciona* and non-chordate invertebrates has expanded into a gene family that includes *Neurog 1, 2* and *3*; all these genes are important regulators of the subtype-specification and differentiation of neurons located in various regions of both the central and peripheral nervous system [172,173]. During mouse neurogenesis, around the E12 stage, *Neurog 1* and *2* are expressed in the ventricular zone of the ventral midbrain, the territory where mesencephalic dopaminergic neurons develop [174,175], while *Neurog 3* expression is seen in neurons adjacent to the floorplate [173]. Loss-of-function experiments revealed that *Neurog 2* affects the generation of dopaminergic neurons in the ventricular and intermediate zone of the ventral midbrain without affecting other types of neurons, demonstrating that *Neurog 2* is an essential regulator required for the differentiation of neural progenitors (*Sox2*^+^ cells) into dopaminergic neuron precursors [174,175]. The fact that *Neurog* is expressed in the dopaminergic neurons of the *Ciona* nervous system suggests that its function in the specification of these neurons is conserved across divergent chordates.

Three additional group A bHLH genes with expression in the SV are Atonal bHLH transcription factor 8 (*Atoh8*), Achaete-scute family bHLH transcription factor.c (*Ascl.c*), and Heart and neural crest derivatives expressed-related (*Hand-r*). *Atoh8* is broadly expressed in the central SV territory and overlaps in part with the ventral expression territory of *Ascl.c* and *Hand-r* (Figure 3B). The expression territories of *Ascl.c* and *Hand-r* also intersect, and partly overlap with those of *Ptf1a*, *Ptf1a-r*, and *Tcf3*, the presumptive *Ciona* PTF1 complex (Figure 3B) [147,165]. Of the other two *Ascl* genes in *C. robusta*, *Ascl.a* is expressed in epidermis and presumptive palps [50]; *Ascl.b* is activated in the dorsal ectoderm by the homeodomain TF Msx, and together with Tox, a high-mobility group TF, controls the differentiation of epidermal sensory neurons [163].

### 6.2. Group B

The genome of *C. robusta* contains 10 genes encoding for bHLH TFs of the phylogenetic group B, which recognize a different set of E-box sites (CACGTG or CATGTTG) [150,176] (Table 2). Genes of this group encode for the most prevalent type of bHLH proteins in animals [148,177]. A number of TFs within this group contain a leucine-zipper dimerization domain contiguous to the HLH domain, and can act as either transcriptional activators or repressors [148,150,176]. WMISH expression analysis revealed that seven bHLH genes of group B are expressed in the SV at the tailbud stages, namely *AP4, Mad, Mitf, Mnt-r, Myc, SREBP,* and *Usf* (Table 1). Most of them exhibit a broad expression in this territory, whereas *Mitf* is localized to the pigmented cells of the ocellus and otolith (Figure 3B) [178]. The expression of *Myc* coincides with that of *Pitx* in the neurohypophyseal duct, and it extends to the anterior-most part of the SV (Figure 3A,B).

*Mad* was previously described as a notochord gene in a screen aimed at the identification of transcriptional targets of the T-box TF Brachyury, and was originally named *Noto7* [179]. In addition to being expressed in the notochord, this gene is expressed in SV, tail epidermis, and muscle [50]. Its expression in the notochord, and its dependence upon Brachyury, suggest that *Mad* might be part of the *Ciona* notochord gene regulatory network, similarly to *Bhlh-tun1* [12,166] and possibly *Ci-ARNT*, another Brachyury-downstream bHLH gene, which is reportedly expressed in the notochord before metamorphosis and is detected in several tissues of juveniles, including the neural complex [136,180] (Table 1). Expression of *Mad* in the developing CNS seems dynamic, as its transcripts are initially detected throughout the periphery of the SV, while the hybridization signal in larvae is less clear and seems more concentrated to the anteriormost region of the SV [50]. This latter pattern seems confirmed by the enhancer activity of a genomic region upstream of the *Mad* transcription start site (our unpublished results).

No expression data are available for *Figla-r*. In vertebrates, *Figla* (Folliculogenesis-specific bHLH transcription factor) is one of the transcription factors exclusively expressed in germ cells, and is active during early folliculogenesis [181,182].

### 6.3. Group C

The bHLH TFs of Group C contain, in addition to the bHLH domain, the Period-ARNT-Single-minded (PAS) domain, which can be present in either single or multiple copies [147,150]. The PAS domain consists of ~260–310 amino acids and is required for dimerization between PAS-containing proteins, for interactions with non-PAS proteins, and for binding to small molecules, such as dioxin [183,184]. This domain also functions as a signaling sensor that monitors changes in light, oxygen, redox potential, and overall energy levels in the cell [150,185]. In the genome of *C. robusta* there are five genes that encode for bHLH-PAS proteins, namely *Hif*, *ARNT, Sim*, *AHR,* and *Trh*, all with respective counterparts in vertebrates [50,147] (Table 2).

*Ciona Hif* (*Ciona hypoxia inducible factor*, counterpart of vertebrate *Hif1α*) encodes a bHLH-PAS TF that is ubiquitously expressed throughout early *Ciona* embryogenesis, and later, during the tailbud stages, becomes restricted to mesenchyme and nervous tissue within the trunk [30]. Moret et al. [30] also demonstrated that in *Ciona* tailbuds *Hif* is expressed in the anterior and ventromedian neural tube, in a small group of cells nestled between the expression territories of *Otp* and *Meis* (Figure 3B). In vertebrates, ARNT (Aryl Hydrocarbon Receptor Nuclear Translocator) and Sim (Single-minded) are known to be interacting partners of Hif1α, and together, these TFs are required for the differentiation of hypothalamic neuroendocrine cell types of the paraventricular and supraoptic nuclei [30,186]. In *Ciona*, the expression of *ARNT* in the SV is unclear, and that of *Sim* remains to be determined, hence it is not possible to determine whether *Hif* expression is sufficient for the specification and differentiation of any specialized cell types within the SV.

### 6.4. Group D

The bHLH TFs of the phylogenetic group D lack the basic domain preceding the HLH domain and cannot bind to DNA. Still, they are able to form heterodimers with other bHLH TFs and to antagonize their transcriptional activity [147,148]. *C. robusta* contains only two bHLH genes of group D: Inhibitor of DNA binding a (*Id.a)* and Inhibitor of DNA binding b *(Id.b)*. *Id.a* and *Id.b* are both located on chromosome 7 in opposite orientations and are separated by ~9kb. This interval is occupied by three unrelated gene models, which could be indicative of a chromosomal rearrangement [140]. Single-cell transcriptomic profiling suggests that both genes are expressed in the wall of the SV [31] (Table 1). Morpholino-mediated knockdown of Id.a resulted in ectopic expression and upregulation of *Id.a* itself, which suggests a negative autoregulatory feedback, either direct or indirect [63]. Moreover, morpholino-mediated knockdown of *Neurog* resulted in the loss of *Id.a* expression, suggesting that *Id.a* is downstream target of *Neurog* [63].

### 6.5. Group E

The bHLH TFs of group E are characterized by the presence of additional motifs in their C-terminal region: a YRPW (Tyr-Arg-Pro-Trp) motif in the Hey subclass, and a WRPW (Trp-Arg-Pro-Trp) motif in the Hairy and Enhancer of split (E(spl)) subclass [147]. In *C. robusta*, there is only one bHLH gene in the Hey subclass (*Hey*), and three genes that belong to the Hairy/ E(spl) subclass (*Hes.a, Hes.b,* and *Hes.c*) [147]. WMISH results show a weak expression of *Hes.a* in three parallel regions located in the dorsal portion of the SV wall [50]; the region showing the sharpest hybridization signal is depicted in Figure 3B. Expression of *Hes.a* in the SV wall is supported by scRNA-Seq data [31] [Table 1). Expression of *Hes.b* is discontinuous and encompasses all four structures of the larval CNS (Figure 3) [50]; in addition to being expressed in the CNS, *Hes.b* is expressed in the tail epidermis, in sharp dorsal and ventral medio-lateral domains [187]. Remarkably, after metamorphosis, Hes.b participates in the regeneration of the oral siphon (see above) [137].

### 6.6. Group F

The bHLH TFs of the phylogenetic group F are characterized by the presence of a COE (Collier/Olf-1/EBF) domain that is involved in dimerization and DNA binding [188]. Ebf is the only bHLH TF of *C. robusta* that meets the structural requirements of this group. *Ebf* exhibits a broad expression pattern in the SV and motor ganglion at the mid-tailbud stage [50]. The homolog of this gene in *C. elegans*, *unc-3*, is required for the regulation of the terminal differentiating features of cholinergic motor neurons, which suggests that the function of this TF has remained conserved throughout evolution [103]. In further support of this point, the expression of *C. robusta Ebf* is able to compensate for the loss of activity of unc-3 in *C. elegans unc-3* mutants [103]. In addition to its function in the larval CNS of *Ciona*, Ebf is also responsible for establishing the pharyngeal muscle cell fate in mixed-fated cardiopharyngeal precursors, through the activation of another bHLH TF, Mrf, and for excluding the cardiac developmental program from these progenitors [189].

### 6.7. Outgroup

Four of the *Ciona* bHLH genes have been tentatively designated as ‘*tunicate bHLH*’ genes (*Bhlh-tun1-4*) because they seemed to lack identifiable counterparts in animals other than tunicates [147,190]. At least two of these genes are expressed in the SV, *Bhlh-tun1* and *Bhlh-tun2*. *Tunicate bHLH 1* (*Bhlh-tun1*) is expressed in the developing notochord, in the midline epidermis of trunk and tail, and in the SV [50,166,191]. The expression pattern of *Bhlh-tun1* in the SV is quite dynamic, and encompasses various small groups of cells, predominantly located in the wall of the SV [31,166] (our unpublished data). Single-cell RNA profiling results indicate expression in several additional SV cell types, including MHB and pigment cells [27]. Studies of its *cis*-regulatory region suggest that *Bhlh-tun1* is also expressed, in late larval stages, in the stomodeum and in the two atrial siphon primordia (Figure 3A) [166]. Before metamorphosis, Bhlh-tun1 is involved in the formation of the notochord and of the neurogenic midline [166,191]. After metamorphosis, Bhlh-tun1 participates in the formation of the musculature of both oral and atrial siphons [189,192]. The bHLH-tun1 protein consists of only 139 amino acid residues, half of which constitute the basic DNA-binding domain, and it does not group with any of the monophyletic bHLH groupings [147,166]. *Bona fide* orthologs of *Bhlh-tun1* are yet to be identified in vertebrates, which suggests that this might be a tunicate-specific gene. Our laboratory’s results on the DNA-binding properties of Bhlh-tun1 indicate that this short protein efficiently binds in vitro E-boxes with different cores [166]. After performing a subtractive microarray screen, we found that bHLH-tun1 is responsible for the transcriptional regulation of at least two genes expressed in the anteroventral SV, namely *Lhx3/4/5*, which encodes for a TF of the LIM homeobox family, and *Gucy1a1*, whose product is a subunit of the guanylate cyclase enzymatic complex [166]. In humans, mutations in the *LHX3* gene have been associated with combined pituitary hormone deficiency, limited neck rotation, and sensorineural hearing loss [193]. In other vertebrates, *Lhx3* and *Lhx4* have been reported to play redundant roles in the development of the adenohypophysis; mouse embryos harboring homozygous null alleles of *Lhx3* and *Lhx4* display arrested development of Rathke’s pouch during early embryogenesis [194,195]. *Lhx3* is also involved in the specification of motor neurons [193,196]. Studies in chick embryos revealed that *Lhx3* expression is restricted to the medial subdivision of the motor columns of the spinal cord, whose motor neurons extend along the entire rostro-caudal length of the spinal cord and project to the axial muscle [196]. Accordingly, *Ciona Lhx3/4/5* is expressed in the motor ganglion [63].

The guanylate cyclase enzymatic complex catalyzes the conversion of guanosine-5’-triphosphate (GTP) to 3′,5′-cyclic guanosine monophosphate (GMP) and pyrophosphate when stimulated by the gaseous signaling molecule nitric oxide (NO) [197]. The NO signaling pathway regulates the neuronal activity in distinct regions of the hypothalamus [197], and in ascidians is involved in the regulation of metamorphosis [198].

Expression of *tunicate bHLH 2* (*Bhlh-tun2*) is localized to a small region of the ventral SV [108] (Figure 3B). This gene was originally categorized as tunicate-specific; however, current tBLASTN searches detected sequence homology between Bhlh-tun2 and Nhlh1, a group A bHLH TF [199]; we suggest that, in light of its sequence similarities, Bhlh-tun2 could be tentatively assigned to this subdivision of the *Ciona* bHLH family. In mouse embryos, Nhlh1 has been recently described as a possible transcriptional regulator of *Onecut1* in fate-restricted retinal progenitors [200]. In *Ciona*, Onecut is an activator of *Rx* expression in the SV, and controls development of the ocellus and its photoreceptors [201]; in turn, expression of *Onecut* is controlled by Neurog in most of its domains, with the notable exception of the SV [202]. Together, the role of Nhlh1 in mouse and the conservation of the function of Onecut in retinal development in *Ciona* suggest the intriguing possibility that Bhlh-tun2/Nhlh1 might be the still uncharacterized activator of *Onecut* expression.

The expression pattern of *Bhlh-tun3* is still uncharacterized; the hybridization signal for *Bhlh-tun4* was not clearly localized [50].

An additional bHLH gene of uncertain classification is *bHLH-like1* (Table 1), which is related to a bHLH TF called spermatogenesis- and oogenesis-specific bHLH-containing protein 1 (Sohlh1). This bHLH TF is expressed in both male and female mammalian germ cells. Together with other TFs, including the bHLH TFs Neurog3 and Sohlh2, Sohlh1 is part of a gene regulatory network that promotes spermatogonial differentiation in male mammals [203], and is required for oogenesis in females [204].

## 7. Cross-Regulatory Interactions among bHLH Transcription Factors in *Ciona*

Autoregulatory feedback and cross-regulatory interactions among transcription factors are major contributors in the generation of the regulatory state of a gene network [205]. In vertebrate organisms, these interactions link numerous bHLH TFs, often in tissue- and organ-specific cross-regulatory networks, as is the case for the bHLH TFs involved in the specification of the cell types present in the retina [206] or in the cochlea [207]. The results of gene inactivation experiments suggest that cross-regulation between bHLH TFs might be widespread in *Ciona* as well. In the nervous system, MO-mediated knockdown of Neurog resulted in reduced expression of *Ebf* in neural precursors [164]. The down-regulation of *Neurog* also caused an increment of its own mRNA levels, suggesting the existence of a negative autoregulatory loop, either direct or indirect [164]. In bipolar tail neurons, Neurog acts as a positive regulator of *bHLH-tun2* expression [108]; these results suggest that the regulatory relationship between these bHLH TFs might be retained in the SV as well.

In the mesenchyme, the MO-mediated knockdown of *Hand-r* (*NoTrl*) resulted in the down-regulation of both *Twist-r.a* and *Twist-r.b* [164]. Hand-r morphants also exhibited an increment in the mRNA levels of *Hand-r*, which suggests that a negative autoregulatory feedback might occur for this gene as well [164]. In the trunk ventral cells (TVCs), the heart precursors, the expression of *Hand-r* is regulated by another bHLH TF, Mesp [22,160,164]. Mesp morphants also display an increase in *Mesp* transcription [164]. Similar results were observed in Mrf morphants [164], suggesting that negative autoregulatory feedback is a widespread characteristic of bHLH TFs.

Also in cardiopharyngeal precursors, another bHLH TF, Ebf, is able to promote the pharyngeal muscle fate by activating the muscle differentiation program downstream of Mrf [189].

In the epidermis, overexpression studies and results of microarray screens indicate that Bhlh-tun1 down-regulates *Ascl.a* and *Hes.c* [166,191]. In embryos overexpressing Bhlh-tun1 in the notochord, expression of *Bhlh-tun1* itself is reduced, which provides another example of a bHLH TF fine-tuning its own transcription [166].

## 8. Conclusions

Differently from developmental regulators of the homeodomain family, which pattern the body plan along its anterior-posterior and proximal-distal axes across metazoan, transcription factors of the bHLH superfamily are often involved in cell-lineage determination and cell differentiation. In ascidians and vertebrates alike, bHLH TFs are required, in particular, for the regulation of both myogenesis and neurogenesis. During vertebrate neurogenesis, bHLH TFs, which are frequently present in these complex genomes in multiple copies, act in a partially redundant fashion to generate neuronal diversity within the main subdivision of the developing nervous system. Despite being several orders of magnitude less complex than a vertebrate brain, the CNS of *Ciona* expresses at least 21 different bHLH TFs within the roughly 330 neural and non-neural cells that compose its four anatomical compartments. A large fraction of these genes are expressed in partially overlapping territories, and this suggests that their products can form different heterodimers with varying transcriptional activity. In turn, different heterodimers, and the target genes that they control, are potentially able to generate sub-domains of neural gene expression within the *Ciona* CNS. The results of gene expression studies reviewed here suggest that some of these molecular compartments could be regarded as predecessors of structures that are anatomically and physiologically distinguishable in the vertebrate brain, such as hypothalamus and retina.

In addition to the physical interactions shared by different bHLH TFs through the formation of heterodimers, these factors are also largely interconnected at the transcriptional level by cross-regulatory interactions. Consequently, the expansion of the complement of bHLH genes and their respective *cis*-regulatory regions that accompanied vertebrate evolution has not only widened the repertoire of possible bHLH dimers, but has also increased the intricacy of the network of cross-regulatory interconnections that existed in invertebrate chordates. A few examples of these interconnections have already been identified in *Ciona*, and additional ones will likely be discovered as more functional studies of bHLH genes are performed in this organism.

Another mechanism that, during vertebrate evolution, has led to the appearance of structures that were not present in invertebrate chordates, the so-called ‘vertebrate innovations’, is the co-option of existing transcription factors, and their respective downstream genes, to different or novel cell types. Studies in *Ciona* suggest that bHLH TFs that mediate epithelial-mesenchymal transition and cell migration, as well as the bHLH TFs that participate in the melanocyte-specific gene regulatory network, were present in a common chordate ancestor. Their co-option to emerging cell types might have represented a key event in the appearance of migrating neural crest cells and their derivatives, which include the vertebrate craniofacial structures. In a similar way, the increasing intersections among the territories of expression of bHLH TFs, and the expansion of their cross-regulatory network, likely drove the emergence of novel anatomical structures and shaped functional compartments in the vertebrate brain.

**Table 1:** Genes encoding transcription factors of the bHLH family in the ascidian *Ciona robusta*.

Gene Name	Alternative Names	Human Hits *	Gene Models KH2013 KY2019	Expression at Mid/Late Tailbud	Reference	scRNA-Seq Data from Larvae	
						[27]	[31]
*Ascl.a (achaete-scute family bHLH transcription factor.a)*	*Achaete-Scute a-like2*	ASCL1; ASCL3; ASCL5	KH.L9.13 KY.Chr2.2314	Epid., palps	[50]		
*Ascl.b (achaete-scute family bHLH transcription factor.b)*	*Achaete-Scute b*	ASCL3; ASCL4; ASCL5	KH.C2.880 KY.Chr2.2022	ESNs	[163]		
*Ascl.c (achaete-scute family bHLH transcription factor.c)*	*Achaete-Scute a-like1*	ASCL3; ASCL5; NEUROD1	KH.C2.560 KY.Chr2.1484	Anterior SV, weak mesench. and palps	[50]; Figure 3		
*Atoh8 (Atonal bHLH transcription factor 8)*	*Net*; *NeuroD-like*	ATOH1; ATOH8; NEUROD4	KH.C9.872 KY.Chr9.174	Anterior SV, MG, cESNs, trunk endod.	[50,84] Figure 3	aSV	Ventral SV
*Atonal*		ATOH1; ATOH7; NEUROD1	KH.C8.175 KY.Chr8.248	ESNs, palps	[50]	cESNs rTENs	
*Bhlha15 (basic helix-loop-helix family member a15)*	*Mist*	BHLHA15; NEUROG1; NEUROG2	KH.C3.308 KY.Chr3.1309	Mesench.	[50]		
*Hand (heart and neural crest derivatives expressed)*		HAND1; HAND2; TCF15	KH.C14.604 KY.Chr14.359	Mesench.	[50]		
*Hand-r (heart and neural crest derivatives expressed-related)*	*NoTrlc*	HAND1; HAND2; SCX	KH.C1.1116 KY.Chr1.2070	Mesench., TVCs, SV	[164]; Figure 3		
*Mesp (mesoderm posterior bHLH transcription factor)*		MESP2; MSGN1; PTF1A	KH.C3.100 KY.Chr3.993	Anterior ventral primary muscle, TVCs	[159]		
*Mrf (Myogenic regulatory factor)*	*MyoD; CiMDFa*	MYF5; MYF6; MYOD1	KH.C14.307 KY.Chr14.1058	Muscle	[156]		
*Neurog (Neurogenin)*		NEUROG1; NEUROG2; NEUROG3	KH.C6.129 KY.Chr6.427	SV, MG, NC Atrial ectoderm	[50,147,208] Figure 3	aATENs, aSV, MHB, SV, MG, Epend.	Dorsolat. SV; SV wall
*Ptf1a (pancreas associated transcription factor 1a)*	*Ptfa*	not determined	KH.C3.967 KY.Chr3.526	SV	[165]; Figure 3		
*Ptf1a-r (pancreas associated transcription factor 1a-related)*	*Ptfb*	not determined	KH.L116.39 KY.Chr11.543	SV	[50]; Figure 3	Coronet cells	Ventral SV
*Tcf3* *(transcription factor 3)*	*E12/E47 E2A*	not determined	KH.C3.480 KY.Chr3.781	Diffuse signal, predominant in SV ^#^	[50]; Figure 3		
*Tcf15-r (transcription factor 15-related)*	*Paraxis-like*	SCX; TAL2; TCF15	KH.S781.11 KY.Chr11.73	No expression	[160]		
*Twist-r.a (twist family bHLH transcription factor-related.a)*	*Twist-like-1a*	ATOH1; NEUROD6; PTF1A	KH.C5.416 KY.Chr5.356	TLCs	[164]		
*Twist-r.b (twist family bHLH transcription factor-related.b)*	*Twist-like-1b*	ATOH1; NEUROD6; NEUROG2	KH.C5.554 KY.Chr5.355	Mesench.	[124]		
*Twist-r.c (twist family bHLH transcription factor-related.c)*	*Twist-like-2*	TAL2; TWIST1; TWIST2	KH.C5.202 KY.Chr5.357	Mesench., TLCs	[50]		Mesench.
*AP4 (transcription factor AP-4)*		SREBF1; TFAP4; TWIST1	KH.C14.448 KY.Chr14.930	Mesench., SV	[50]; Figure 3		
*Figla-r (Folliculogenesis specific bHLH transcription factor-related)*		FIGLA; NHLH1; TAL2	KH.C2.1152 KY.Chr2.2108	Not analyzed			
*Mad (Mothers against dpp)*	*Noto7*	MXD1; MXD4; MXI1	KH.C1.661 KY.Chr1.761	SV, MG, palps, notochord, tail epid., tail muscles	[50,179]	Neurons, notochord	Dorsolat. SV, ventral SV, Endod., Epid.
*Max (Myc associated factor X)*		Not determined	KH.C5.373 KY.Chr5.121	Mesench.	[50]	ANB, aSV, MG	Epid., Mesench., Endod.
*Mitf (Microphthalmia-associated transcription factor)*		MITF; TFE3; TFEB	KH.C10.106 KY.Chr10.837	Mesench., pigmented cells (otolith, ocellus), MG	[50,120]; Figure 3	Collocytes aATENs	Epid.
*Mlx (Max-like protein x)*		MLXIP; MLXIPL	KH.C11.706 KY.Chr11.477	Mesench.	[50]		
*Mnt-r (Max network transcriptional repressor-related)*	*Mnt-like*	MNT; MXD1; MXD3	KH.L20.34 KY.Chr6.608	No expression; a faint signal in SV, palps and mesench. might be present in late tailbuds	[50]		
*Myc (Myelocytomatosis)*		MNT; MYC; MYCN	KH.L24.23 KY.Chr1.686	Mesench., anterior SV, trunk endod.; in juveniles: endostyle pharyngeal gills, heart, intestine, body wall muscle, neural gland epidermis, stomach, esophagus	[50,145] Figure 3	ANB	Endod., Epid.
*Srebp (Sterol regulatory element-binding transcription factor 1)*		SREBF1; SREBF2	KH.L99.12 KY.Chr9.7	SV, palps; epid. (in larvae)	[50,141] Figure 3	NC	SV wall, ventral SV
*Usf (Upstream transcription factor)*		USF1; USF2; USF3	KH.C3.624 KY.Chr3.1438	Mesench., faint signal in SV and NC	[50]; Figure 3		Dorsolat. SV, Mesench., Epid.
*AHR (Aryl hydrocarbon receptor)*		AHR; AHRR; SIM2	KH.C12.93 KY.Chr12.869	Mesench., palps, NC	[50]	Collocytes aATENs	Epid.
*ARNT (Aryl hydrocarbon receptor nuclear translocator)*		ARNT; ARNT2; ARNTL	KH.C5.213 KY.Chr5.617	Weak mesench., notochord, unclear signal in epidermis; in juveniles: endostyle pharyngeal gills, neural gland, stomach, esophagus	[50,136,180]		
*Hif (Hypoxia inducible factor)*		EPAS1; HIF1A; HIF3A	KH.C4.83 KY.Chr4.583	Mesench., anterior SV, palps, ventral midline, tail epid.; in juveniles: endostyle pharyngeal gills, heart, intestine, body wall muscle, neural gland epidermis, stomach, esophagus	[30,50]; Figure 3	aSV	
*Sim (Single-minded)*		Not determined	KH.L20.56 KY.Chr6.618	Not analyzed			
*Trh (Trachealess)*		NPAS1; NPAS3; SIM2	KH.L154.23 KY.Chr11.674	Mesench., very weak SV	[50]		
*Id.a (inhibitor of DNA binding.a)*	*Emc*	ID2; ID3	KH.C7.692 KY.Chr7.1153	Not analyzed			SV wall, mesench., epid.
*Id.b (inhibitor of DNA binding.b)*	*Emc2*	ID1; ID2; ID3	KH.C7.157 KY.Chr7.1157	SV, MG, NC; palps, tail epid., ESNs	[50]	NC, aSV	SV wall, ventral SV, Endod., Mesench.
*Hes.a (hairy and enhancer of split.a)*	*E(spl)/hairy-a*	HES1; HES2; HES4	KH.C1.159 KY.Chr1.28	Muscle, SV, epid.	[50]; Figure 3	NC	Epid., Mesench., Endod., SV wall, dorsolat. SV
*Hes.b (hairy and enhancer of split.b)*	*E(spl)/hairy-b*	HES1; HES4; HES6	KH.C3.312 KY.Chr3.580	Patchy SV, trunk epid., rows of tail epid.; in juveniles: body wall muscle, stigmatal cells	[50,137,187]	Epend., NC, aSV, MHB, SV	Dorsolat. SV, Epid., SV wall, Endod.
*Hes.c (hairy and enhancer of split.c)*	*E(spl)/hairy-c*	HES1; HES2; HES4	KH.L34.9 KY.Chr1.1234	No distinct zygotic signal, ubiquitous staining throughout embryogenesis	[50]	Collocytes	Endod., epid.
*Hey (hes related family bHLH transcription factor with YRPW motif)*		HEY1; HEY2; HEYL	KH.L130.6 KY.Chr10.1431	No distinct zygotic signal	[50]		Endod., epid., mesench.
*Ebf (Ebf transcription factor)*	*COE*	EBF1; EBF2; EBF3	KH.L24.10 KY.Chr1.724	SV, MG; neurohypophysis primordium; tail epid.	[50]; Figure 3	Epend., Eminens cell, pSV aSV, MG	Dorsolat. SV
*bHLH-like1*		CCDC169-SOHLH2; SOHLH1; SOHLH2	KH.C9.380 KY.Chr9.350	Not analyzed			
*Bhlh-tun1* *(Tunicate bhlh 1)*	*Orphan bHLH-1*	Not determined	KH.C7.269 KY.Chr7.1158	SV, palps, notochord, epid.	[50,166]; Figure 3	NC, aSV, PSCs related, pigment cells, MHB, SV, pSV, Epend., Notochord	SV wall, ESNs, ventral SV, epid., endod., mesench.
*Bhlh-tun2* *(Tunicate bhlh 2)*	*Orphan bHLH-2*	NHLH1; NHLH2; TAL2	KH.C4.649 KY.Chr4.1008	SV, BTNs, mesench.	[50,108]; Figure 3	aSV, MG, Eminens cell	Dorsolat. SV
*Bhlh-tun3* *(Tunicate bhlh 3)*	*Orphan bHLH-3*	Not determined	KY.Chr10.1238	Not analyzed			
*Bhlh-tun4* *(Tunicate bhlh 4)*	*Orphan bHLH-4*	Not determined	KH.L41.39 KY.Chr4.1211	No distinct zygotic signal	[50]		
*Tcf4* *(transcription factor 4)*		TFDP1; TFDP2; TFDP3	KH.L60.12 KY.Chr1.10	No distinct zygotic signal; weak mesench. throughout embryogenesis	[50]		

* Retrieved from the Aniseed database [209]. ^#^ From the Ghost database [210]. Abbreviations: aATENs, anterior apical trunk epidermal neurons; ANB, anterior neural boundary; aSV, anterior sensory vesicle; BTNs, bipolar tail neurons; cESNs, caudal epidermal sensory neurons; DEGs, differentially expressed genes; dorsolat., dorsolateral; endod., endoderm; epend., ependymal cells; epid., epidermis; ESNs, epidermal sensory neurons; mesench., mesenchyme; MG, motor ganglion; MHB, midbrain–hindbrain boundary; NC, nerve cord; rTENs, rostral trunk epidermal neurons; SV, sensory vesicle; TLCs, trunk lateral cells; TVCs, trunk ventral cells.

**Table 2:** Classification of bHLH genes in the ascidian *Ciona robusta.* Modified from [147,148].

Phylogenetic Group	Characteristics	*C. robusta* bHLH Genes
A	Bind to CAGCTG or CACCTG	Ascl.a, Ascl.b, Ascl.c *, Atoh8 *, Atonal, Bhlha15, Hand, Hand-r *, Mesp, Mrf, Neurog *, Ptf1a *, Ptf1a-r *, Tcf3 *, Tcf15-r, Twist-r.a, Twist-r.b, Twist-r.c
B	Bind to CACGTG or CATGTTG	AP4*, Figla-r ^#^, Mad *, Max, Mitf *, Mlx, Mnt-r *, Myc *, Srebp *, Usf *
C	Bind to ACGTG or GCGTG. Contain a PAS domain	AHR, ARNT, Hif *, Sim ^#^, *Trh*
D	Lack basic domain and do not bind to DNA. Act as antagonists of group A bHLH proteins	Id.a ^#^, Id.b *
E	Contain an orange domain and a WRPW peptide	Hes.a *, Hes.b *, Hes.c, Hey
F	Contain an additional COE domain, which is involved in dimerization and DNA binding	Ebf *
Outgroup		Bhlh-tun1 *, Bhlh-tun2 *, Bhlh-tun3 ^#^, Bhlh-tun4 Uncertain classification: *bHLH-like1 ^#^*

* Genes that are expressed in the sensory vesicle territory at the mid/late tailbud stage and/or in the adult neural complex, as determined by whole-mount in situ hybridization. ^#^ Genes without published whole-mount in situ hybridization data.

## Abbreviations

ANR	anterior neural ridge;
bHLH	basic helix-loop-helix
BTN	bipolar tail neuron
CNS	central nervous system;
FGF	Fibroblast growth factor
GMP	3′,5′-cyclic guanosine monophosphate
GTP	guanosine-5′-triphosphate
hr(s)	hour(s);
kb	kilobase(s), or 1000 base pairs;
MG	motor ganglion;
MHB	midbrain-hindbrain boundary;
MO	morpholino oligonucleotide;
NC	nerve cord;
SV	sensory vesicle;
TF(s)	transcription factor(s);
WMISH	whole-mount in situ hybridization;
ZLI	zona limitans intrathalamica.
